# Supplementary material for: Non-contact identification and differentiation of illicit drugs using fluorescent films
Source: Nat Commun. 2018 Apr 27;9:1695. doi: 10.1038/s41467-018-04119-6 (PMC5923207; doi:10.1038/s41467-018-04119-6)
Supplement: Supplementary file 1 — Supplementary Information [file 41467_2018_4119_MOESM1_ESM.pdf]

Non-contact Identification and Differentiation of Illicit Drugs Using Fluorescent Films

(Liu et al.)

Supplementary Information

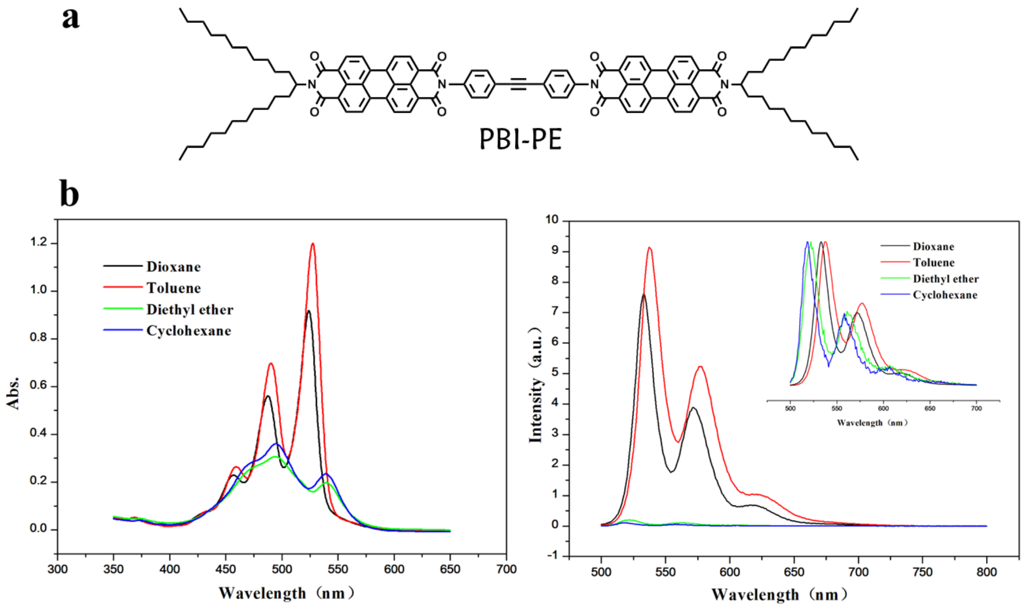

Supplementary Fig. 1 Structure of PBI-PE and its spectroscopic properties in different solvents. **a** Chemical structure of PBI-PE. **b** UV-vis absorption and emission spectra of PBI-CB recorded at a concentration of 5.0×10^{-6} mol L^{-1} and at room temperature in different solvents (λ_{ex}=480 nm).

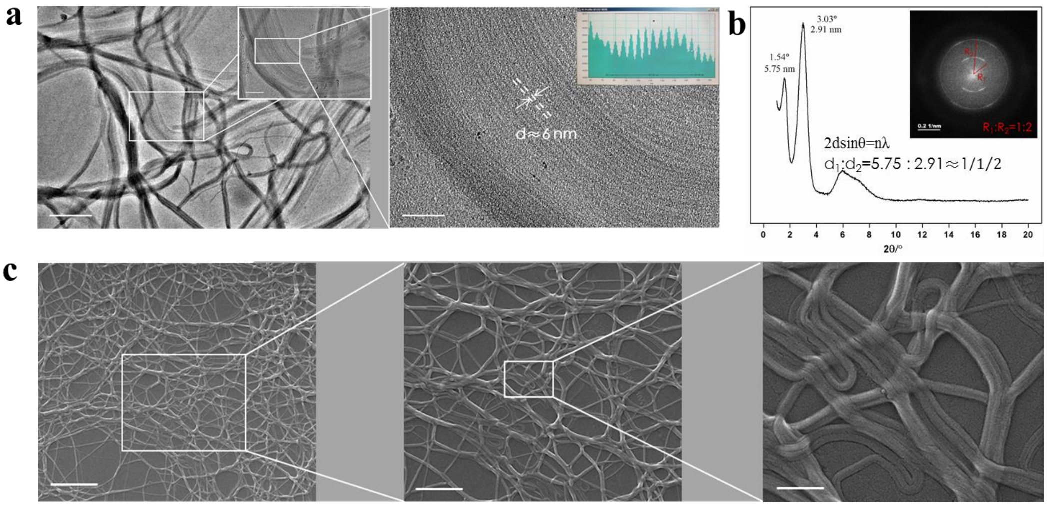

Supplementary Fig. 2 Fibrous structures of PBI-CB aggregates. **a** TEM images with different magnifications (scale bars: left, 400 nm; right, 50 nm; inset of left picture, 100 nm). **b** Relevant electron diffraction pattern, and X-ray diffraction trace of the PBI-CB aggregates. **c** SEM images of the PBI-CB aggregates obtained after 3 hours self-assembly in a mixed solvent of dioxane and water (92.5:7.5, volume ratio) at a concentration of 5×10^{-5} mol L^{-1} (Scale bars: left, 5 μm; middle, 2.5 μm; right, 0.5 μm).

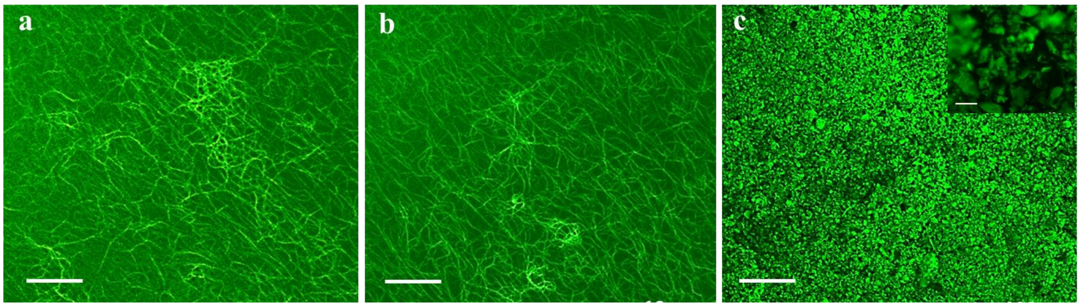

Supplementary Fig. 3 Fluorescence microscope images of the films. Note: (1) **a**, **b** and **c** stand for Film 1, Film 2 and Film 3, respectively; (2) The fluorescence images were obtained under visible light illumination (488 nm); (3) Scale bars: 10 μm (a, b and the inset of c), 100 μm (c).

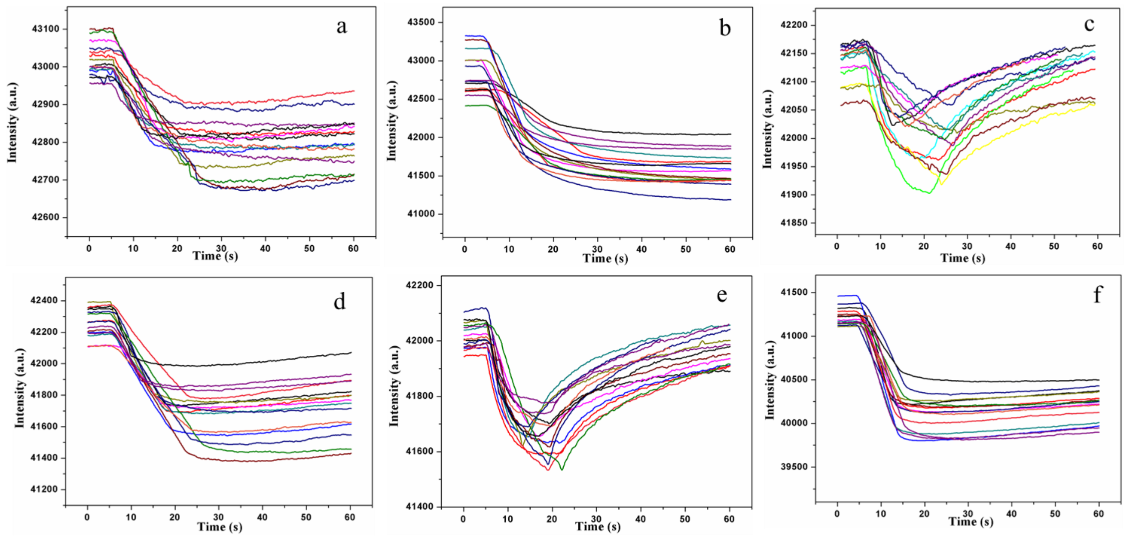

Supplementary Fig. 4 Dynamic response traces of Film 1. Note: (1) Fifteen measurements were conducted for each sample, of which the tests were made by performing blank test first (base line), then a drug vapor (saturated) was sampled, and then the sample was removed; (2) For each measurement, the fluorescence intensity of the film was monitored throughout the three processes; (3) The illicit sample employed are MAPA (**a**), Magu (**b**), Ketamine (**c**), Ecstasy (**d**), Phenobarbital (**e**) and Caffeine (**f**); (4) The sampling time and the distance between the sample and the sampling nozzle were not strictly controlled.

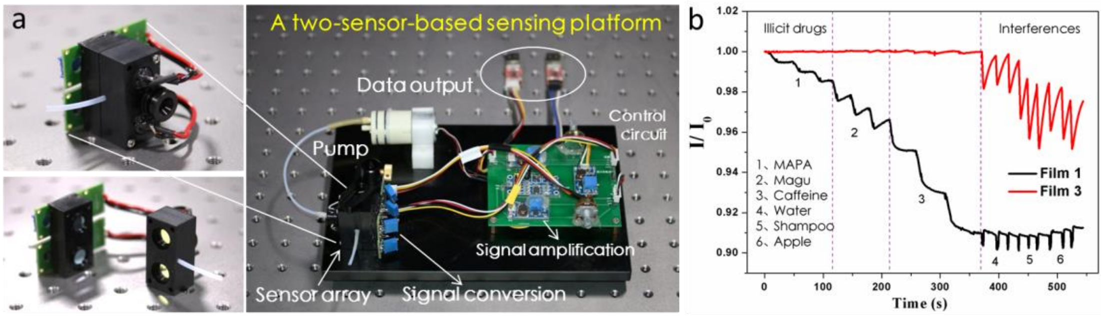

Supplementary Fig. 5 Two-sensor based sensory sensing. **a** Film 1 and Film 3 based 2×1 (two different sensors) sensor array and related sensing platform. **b** Results obtained from three repetitive measurements of three typical illicit drugs and three potential interferences by using the sensor array.

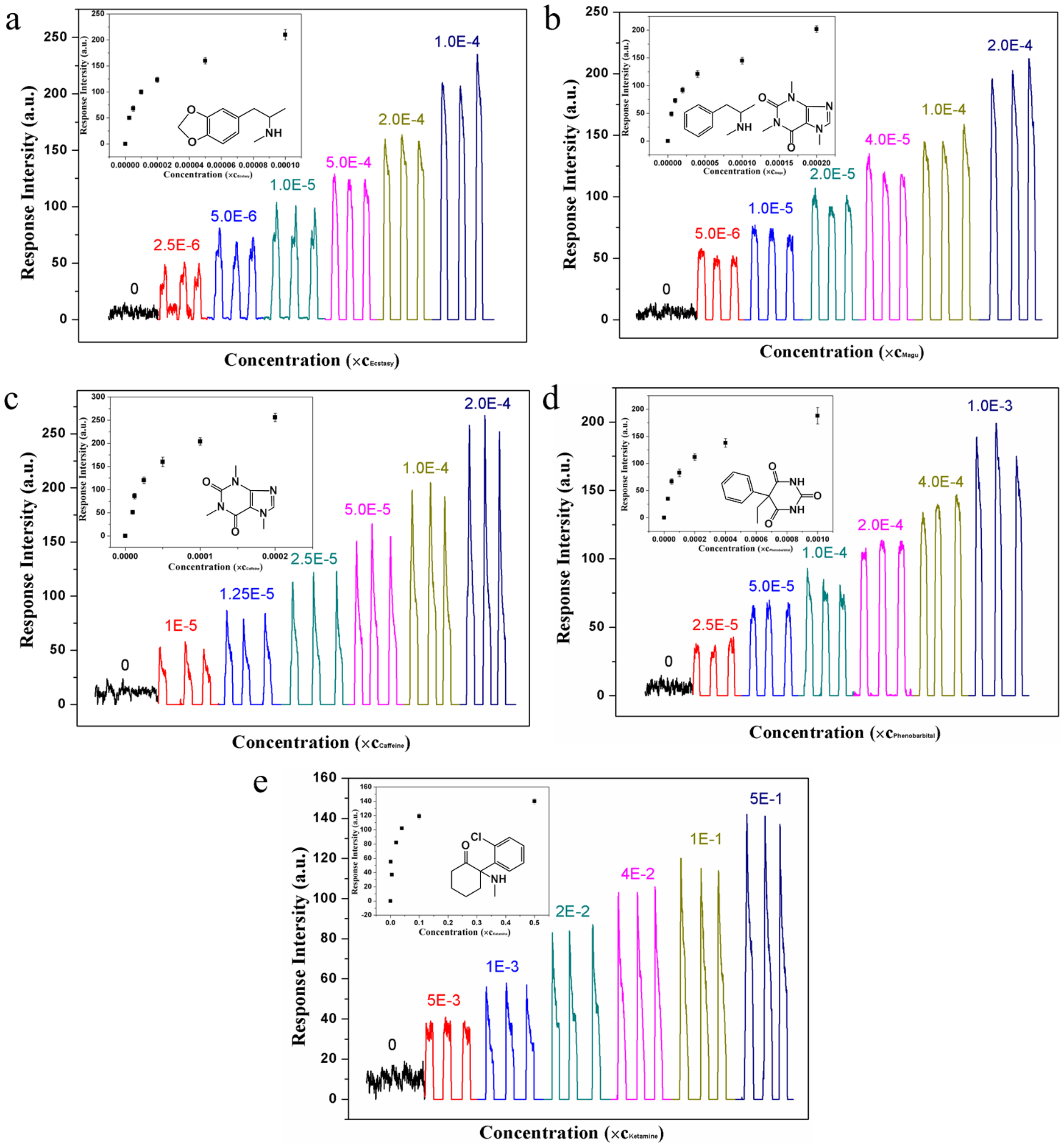

Supplementary Fig. 6 Computer displayed results from the measurements. Note: (1) Film 1 was employed as the sensing film; (2) The numbers on the topside of the traces are the dilution times of the saturated vapors of the illicit drugs, which are ecstasy (**a**), magu (**b**), caffeine (**c**), phenobarbital (**d**), and ketamine (**e**), respectively; (3) The measurements were conducted on the homemade sensing platform depicted in Fig. 4b (main text); (4) Each measurement was repeated five times and the error bars shown in the insets of the figures are drawn from the maximum to the minimum responses.

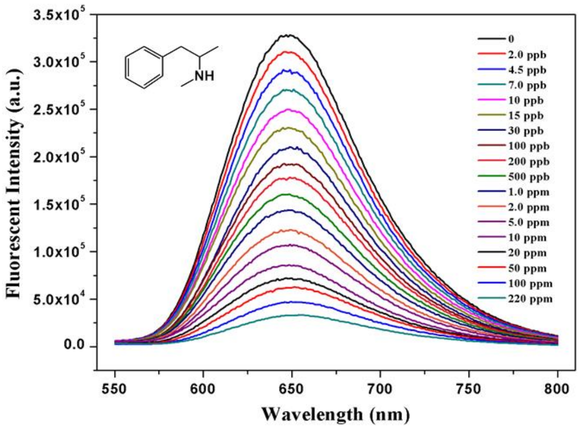

Supplementary Fig. 7 Response results obtained from a commercial instrument. Note: Fluorescence emission spectra of Film 1 recorded in the presence of different concentrations of MAPA vapor, which was generated from dilution of the saturated vapor of MAPA (the vapor pressure of MAPA is ~220 ppm at 25 °C, ref. 4).

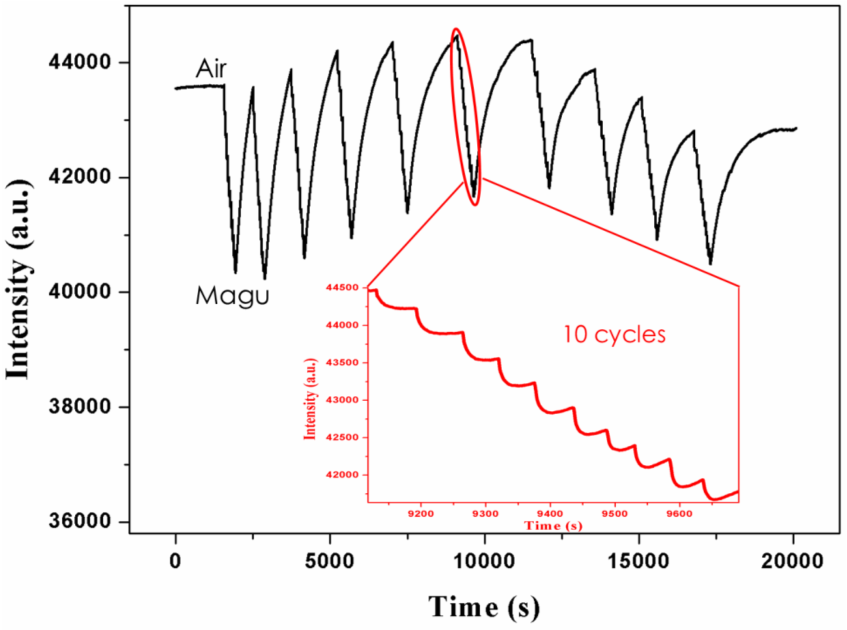

Supplementary Fig. 8 Reusability and reversibility tests. Note: (1) Film 1 was adopted as the sensing film, and magu was taken as a representative illicit drug; (2) To be reliable, more than 100 repetitive tests were conducted. Specifically, to exam the reusability, 10 successive measurements, of which each lasted for 1 minute, were performed first. Then, the sample chamber was purged with air for more than 10 minutes to examine if the emission is recoverable. As depicted in the traces, full recovery is obviously seen, confirming the recoverability and reusability of the film.

Supplementary Table 1 Discrimination and identification of illicit drugs. **a** The truth table of the responses recorded as shown in Supplementary Fig. 5b, where positive response no matter quenching or sensitizing is counted as ′1′, and no response as ′0′. **b** The logic gates formed with the two films.

Film 1	Film 3	Analytes
0	0	No
1	0	Illicit Drugs
1	1	Interferences

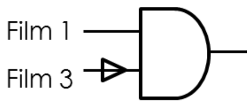

$$F = A \cdot \overline{B}$$

A	B	F
0	0	0
1	0	1
1	1	0

Supplementary Table 2 R values for each illicit drug sample from the fifteen repetitive tests calculated by using equation 1.

	**MAPA**	**Ecstasy**	**Caffeine**	**Magu**	**Ketamine**	**Phenobarbital**
	-0.041	-0.108	-0.137	0.334	-0.977	-0.823
	0.005	-0.147	-0.079	0.311	-0.817	-0.896
	-0.088	-0.102	-0.095	0.469	-1.030	-0.822
	-0.052	-0.125	-0.087	0.603	-1.061	-1.041
	-0.132	-0.116	-0.061	0.437	-1.271	-0.85
	-0.111	-0.055	-0.107	0.517	-0.832	-0.866
	-0.038	-0.015	-0.104	0.478	-0.852	-0.867
**R**	-0.039	-0.043	-0.042	0.594	-0.823	-0.910
	-0.015	-0.185	-0.078	0.306	-0.904	-0.846
	-0.038	-0.002	0.015	0.356	-1.065	-0.809
	-0.051	-0.073	-0.028	0.491	-1.063	-0.957
	0.093	-0.114	-0.051	0.418	-0.927	-0.971
	0.082	-0.164	-0.020	0.563	-1.050	-0.961
	0.009	-0.187	-0.015	0.658	-0.982	-1.004
	-0.184	-0.192	0.093	0.413	-0.894	-0.856
	**-0.2<R<0.1**			**R>0.3**	**R<-0.8**	

Supplementary Table 3 A_1 and A_2 values for illicit drugs (type 1) from the fifteen repetitive tests calculated by using equation 2.

	***A*₁**	**R²**		***A*₁**	**R²**		***A*₁**	**R²**
**Methamphetamine**	-1.196	0.998	**Ecstasy**	-4.933	0.997	**Caffeine**	-17.42	0.997
	-1.885	0.995		-4.664	0.985		-11.22	0.999
	-2.146	0.996		-4.744	0.997		-17.98	0.999
	-1.188	0.894		-4.075	0.997		-15.62	0.999
	-2.771	0.996		-3.864	0.999		-12.38	0.997
	-1.742	0.998		-5.795	0.994		-10.29	0.999
	-1.304	0.994		-6.505	0.991		-10.45	0.999
	-1.927	0.998		-4.669	0.999		-13.46	0.997
	-0.832	0.986		-3.422	0.997		-15.98	0.998
	-2.129	0.997		-4.002	0.999		-9.702	0.999
	-1.614	0.973		-4.248	0.999		-10.37	0.997
	-2.656	0.993		-3.164	0.995		-13.98	0.993
	-2.080	0.996		-4.865	0.995		-16.20	0.997
	-1.211	0.953		-5.397	0.994		-15.55	0.996
	-2.225	0.990		-5.012	0.982		-7.781	0.998
	**A_2**	**R^2**		**A_2**	**R^2**		**A_2**	**R^2**
	0.0151	0.824		0.2274	0.993		0.3512	0.997
	-0.0182	0.752		0.2752	0.988		0.2943	0.972
	0.0557	0.862		0.1933	0.908		0.4232	0.993
	0.0141	0.868		0.1936	0.955		0.3313	0.986
	0.0817	0.796		0.1345	0.927		0.2510	0.975
	0.0867	0.832		0.0804	0.765		0.2639	0.990
	0.0418	0.831		0.0472	0.856		0.3349	0.964
	0.1127	0.822		0.1149	0.827		0.1979	0.922
	0.0907	0.885		0.1714	0.985		0.3433	0.981
	0.0596	0.802		0.1227	0.784		0.3310	0.831
	0.0513	0.732		0.1538	0.833		0.2520	0.947
	-0.0592	0.782		0.2064	0.906		0.3153	0.988
	-0.0724	0.898		0.1585	0.937		0.2155	0.832
	-0.0161	0.808		0.1691	0.875		0.3764	0.983
	0.0891	0.936		0.2373	0.972		0.3454	0.854

Supplementary Table 4 A and *B* values for illicit drugs (type 3) from the fifteen repetitive tests calculated by using equation 3.

	A	*B*	R^2		A	*B*	R^2
**Phenobarbital**	338	-0.020	0.997	**Ketamine**	236	-0.031	0.998
	368	-0.026	0.999		206	-0.031	0.999
	338	-0.034	0.998		274	-0.019	0.997
	475	-0.028	0.997		234	-0.025	0.997
	536	-0.011	0.998		217	-0.025	0.997
	548	-0.017	0.998		194	-0.013	0.980
	579	-0.020	0.996		190	-0.010	0.993
	416	-0.024	0.971		178	-0.017	0.996
	483	-0.019	0.997		207	-0.023	0.974
	463	-0.009	0.999		178	-0.017	0.996
	369	-0.034	0.990		152	-0.021	0.974
	314	-0.030	0.995		186	-0.028	0.977
	369	-0.028	0.995		213	-0.024	0.983
	329	-0.031	0.997		217	-0.025	0.997
	464	-0.027	0.999		250	-0.014	0.992

Supplementary Methods

Synthesis of geometrical dimer PBI-CB

The structure and synthesis route of PBI-CB are shown in Supplementary Fig. 9.

Compound **1** was synthesized according to literature methods.[1]

Compound **2** was synthesized according to literature methods.[2]

Compound **3** was synthesized according to literature methods.[3]

Synthesis of PBI-PE

A mixture of compound **2** (1.0 g, 1.40 mmol), compound **3** (0.15 g, 0.72 mmol), imidazole (5.0 g), and $Zn(OAc)_2$ (0.25 g) were stirred 2 h at 140 °C. The reaction mixture was cooled to room temperature, dispersed in 30 mL water, and the precipitation was filtered and washed several times with water. Further purification was carried out by column chromatography on silica gel using dichloromethane/acetone (*V*/*V*, 50:1) to obtain the red solid. ^{1}H NMR ($CDCl_3$/TMS, 600 MHz, ppm): δ 8.69 (dd, J = 56.5, 7.3 Hz, 8H, perylene), 7.77 (d, J = 8.3 Hz, 2H, Ph), 7.40 (d, J = 13.3 Hz, 2H, Ph), 5.19 (m, 1H, -C*H*-), 2.26 (m, 2H, -CH_2-), 1.88 (m, 2H, -CH_2-), 1.32-1.20 (m, 36H, -$(CH_2)_{18}$-), 0.84 (t, J = 7.1 Hz, 6H, -CH_3). MS (MALDI-TOF, m/z): Calcd. for [$(M+H)^+$]: 1600.90, found: 1601.46.

Synthesis of PBI-CB

A mixture of decaborane ($B_{10}H_{14}$, 0.045 g, 0.37 mmol) and *N*,*N*-dimethylaniline (0.135 g, 1.11 mmol) in 30 mL of distilled toluene was stirred at R.T. for 30 min under N_2 atmosphere, and then the temperature was raised to 100 °C for 2 h. After cooling down to 40 °C, PBI-PE (0.3 g, 0.19 mmol) was added and the final mixture was refluxed for 10 h. The mixture was cooled to room temperature and quenched with methanol. The solvent was removed under reduced pressure and the residue was purified by silica gel column chromatography using dichloromethane/acetone (*V*/*V*, 50:1) as the eluent. PBI-CB was obtained as a red powder. ^{1}H NMR ($CDCl_3$/TMS, 600 MHz, ppm): δ 8.54 (d, J = 7.8 Hz, 4H, perylene), 8.34 (d, J = 12.1 Hz, 4H, perylene), 7.64 (d, J = 8.4 Hz, 2H, Ph), 7.31 (d, J = 8.4 Hz, 2H, Ph), 5.15 (m, 1H,

-C*H*-), 3.62-1.78 (m, 9H, -C*H*$_2$-, -C*H*$_2$-, -B*H*), 1.34-1.20 (m, 36H, -(C*H*$_2$)$_{18}$-), 0.84 (t, J = 7.1 Hz, 6H, -C*H*$_3$). ^{11}B NMR ($CDCl_3$/TMS, 600 MHz, ppm): δ -3.72, -13.05. ^{13}C NMR ($CDCl_3$/TMS, 600 MHz, ppm): δ 162.91, 136.94, 134.93, 133.70, 131.51, 131.03, 129.52, 129.27, 128.97, 126.03, 123.12, 122.89, 122.75, 84.16, 54.97, 32.36, 31.90, 29.63, 29.62, 29.58, 29.56, 29.32, 27.08, 22.66, 14.08. FTIR (KBr, cm^{-1}): 2929, 2854, 2589, 1710, 1656, 1597, 1516, 1467, 1435, 1397, 1338, 1252, 1171. MS (MALDI-TOF, m/z): Calcd. for [(M+Na)$^+$]: 1741.07, found: 1740.83.

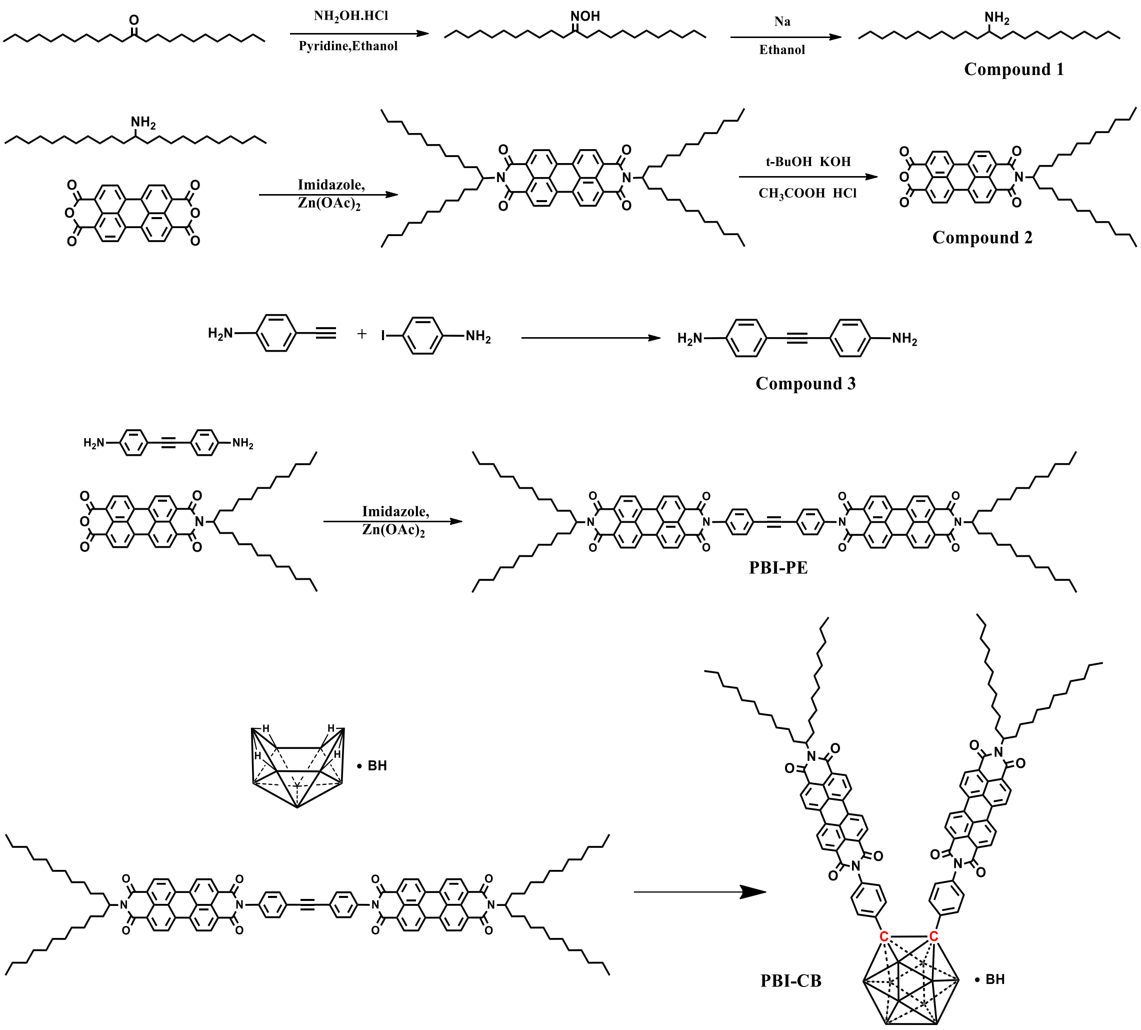

Supplementary Fig. 9 The synthesis route of PBI-CB.

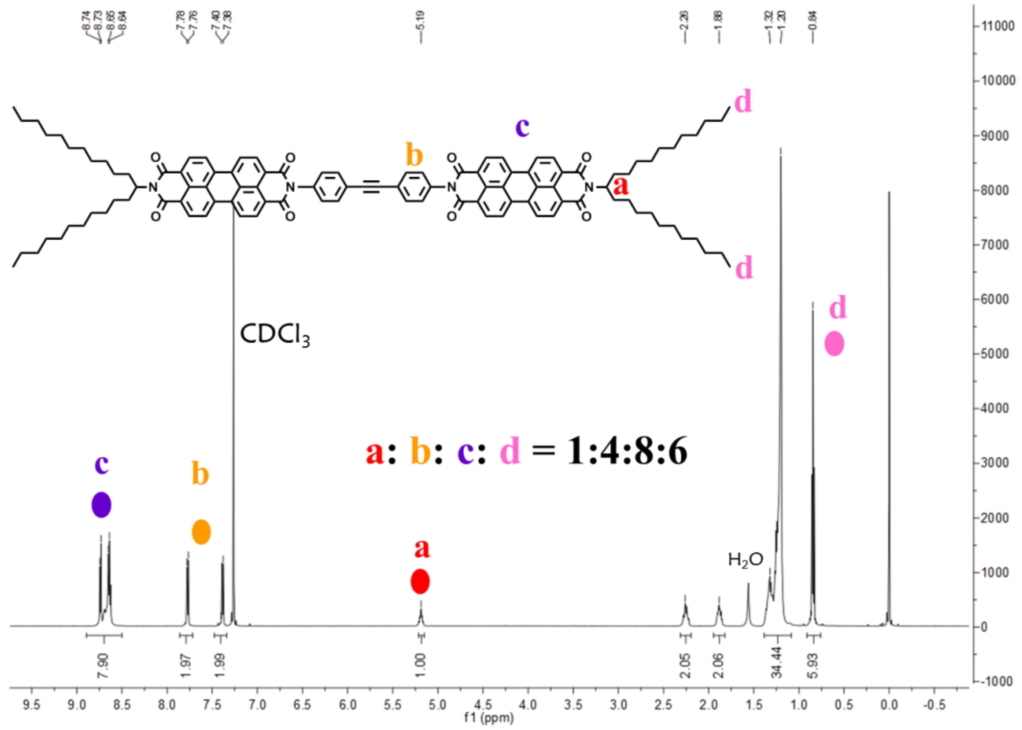

Supplementary Fig. 10 ^{1}H NMR spectrum of PBI-PE in $CDCl_3$.

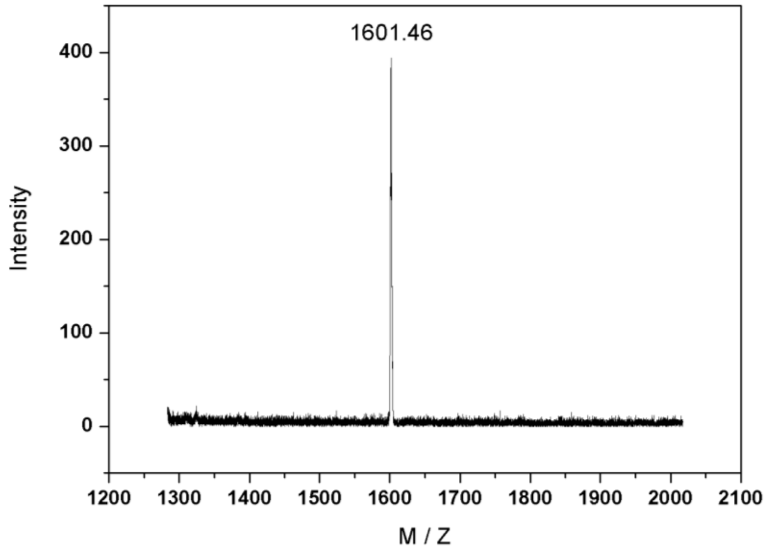

Supplementary Fig. 11 MS spectrum of PBI-PE.

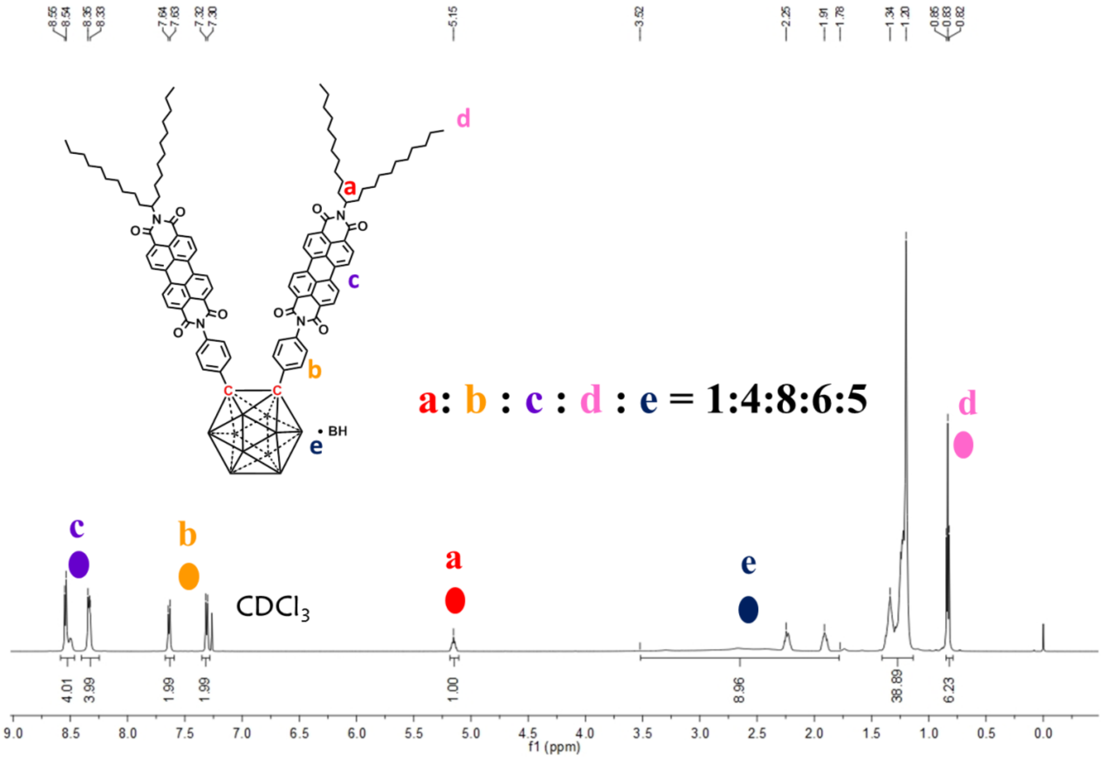

Supplementary Fig. 12 ^{1}H NMR spectrum of PBI-CB in $CDCl_3$.

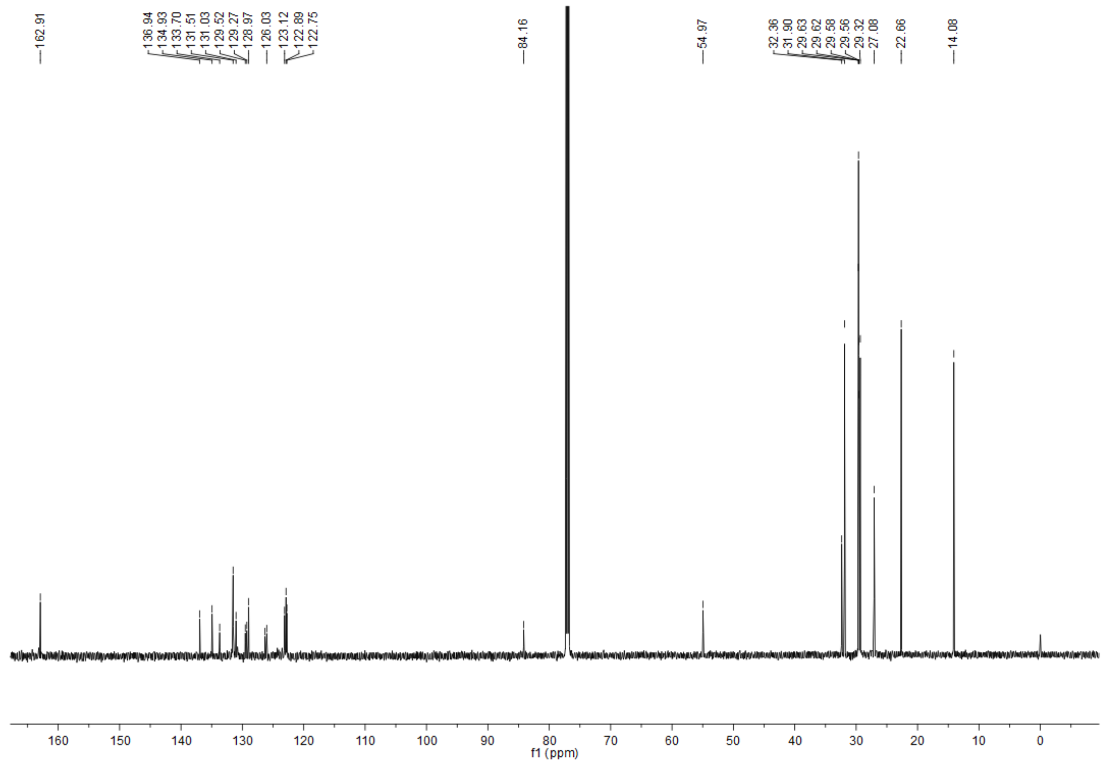

Supplementary Fig. 13 ^{13}C NMR spectrum of PBI-CB in $CDCl_3$.

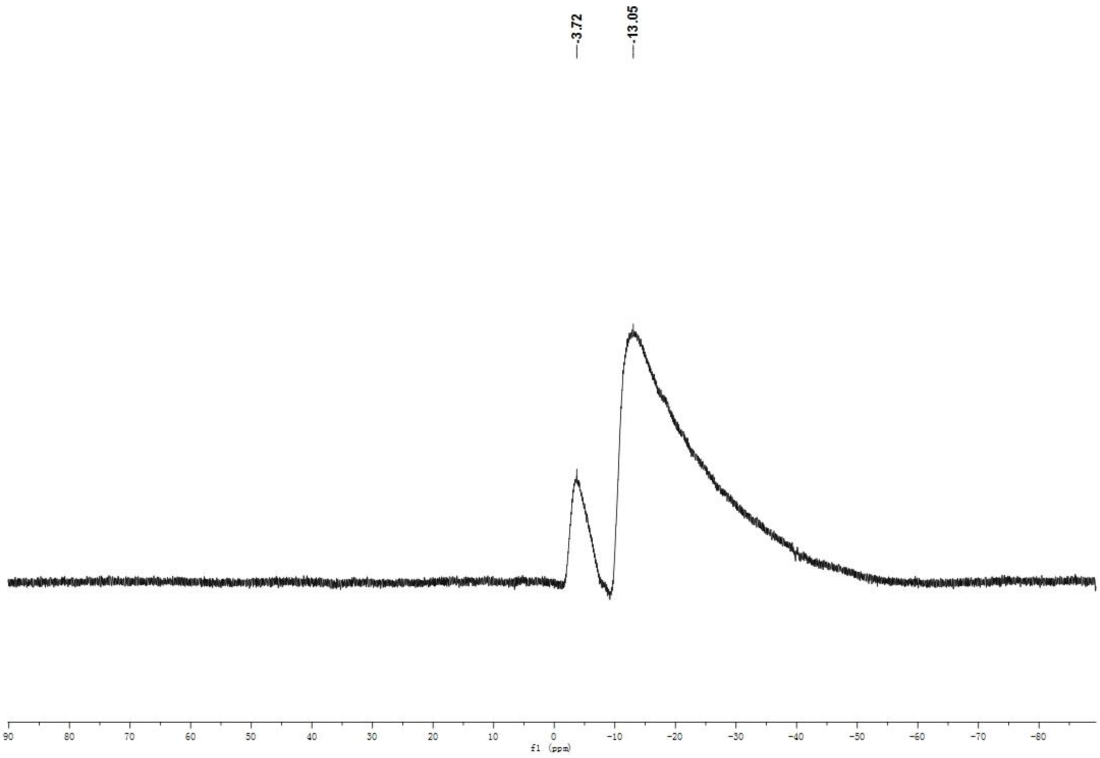

Supplementary Fig. 14 ^{11}B NMR spectrum of PBI-CB in $CDCl_3$.

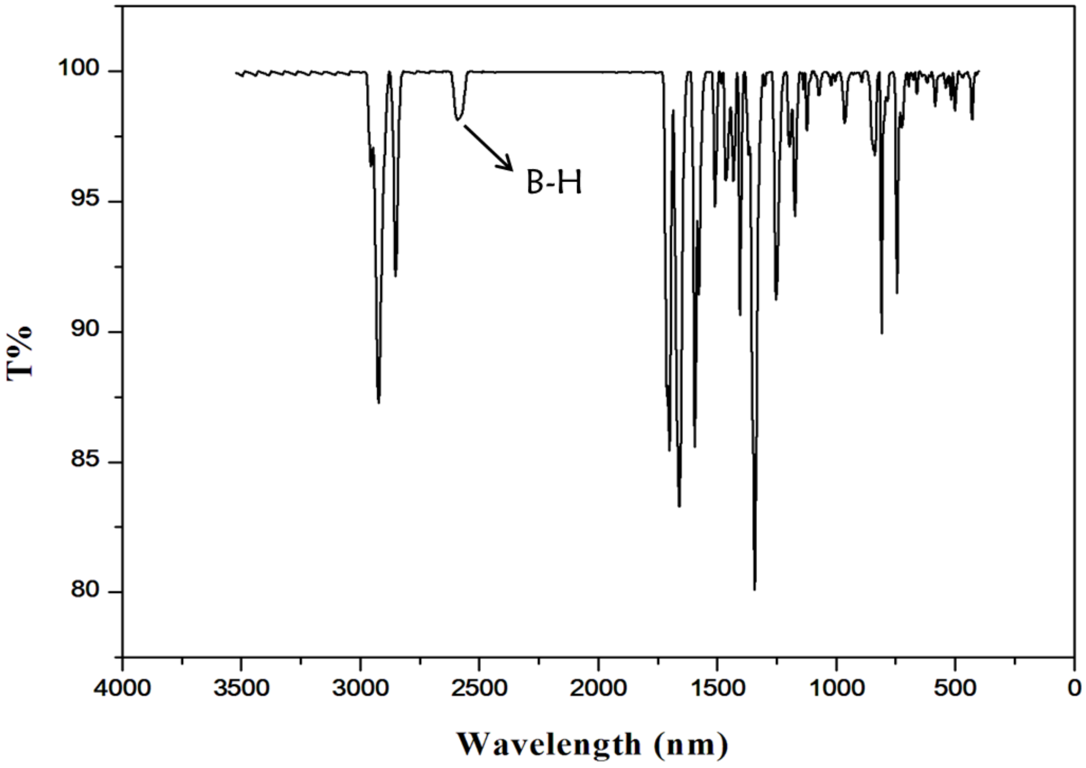

Supplementary Fig. 15 FTIR spectrum of PBI-CB.

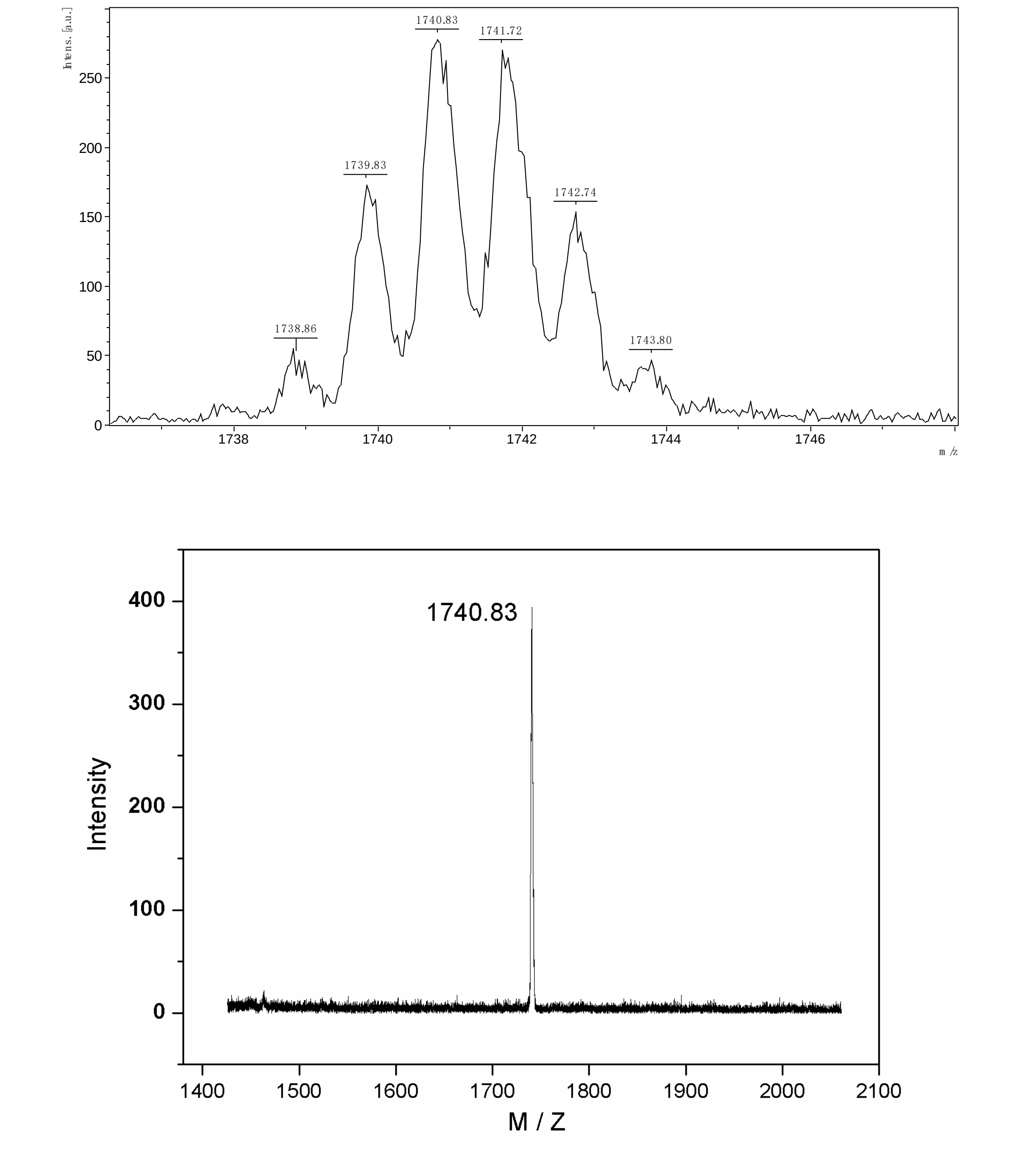

Supplementary Fig. 16 MS spectrum of PBI-CB.

Supplementary References

1. Yuan, Z., Xiao, Y., Yang, Y., & Xiong, T. Soluble ladder conjugated polymer composed of perylenebisimides and thieno[3,2-b]thiophene (LCPT): a highly efficient synthesis via photo-cyclization with the sunlight. *Macromolecules* **44**, 1788-1791 (2011).
2. Lu, C., Fujitsuka, M., Sugimoto, A., & Majima, T. Unprecedented intramolecular electron transfer from excited perylenediimide radical anion. *J. Phys. Chem. C* **120**, 12734-12741 (2016).
3. Nishimura, D. *et al.* Relative rotational motion between γ-cyclodextrin derivatives and a stiff axle molecule. *J. Org. Chem.* **73**, 2496-2502 (2008).
4. Wen, D. *et al.* Fine structural tuning of fluorescent copolymer sensors for methamphetamine vapor detection. *Sens. Actuators B* **168**, 283-288 (2012).
